# Cortical Representation of Species-Specific Vocalizations in Guinea Pig

**DOI:** 10.1371/journal.pone.0065432

**Published:** 2013-06-13

**Authors:** Daniel Šuta, Jiří Popelář, Jana Burianová, Josef Syka

**Affiliations:** 1 Department of Auditory Neuroscience, Institute of Experimental Medicine, Academy of Sciences of the Czech Republic, Prague, Czech Republic; 2 Department of Medical Biophysics and Informatics, Third Faculty of Medicine, Charles University in Prague, Prague, Czech Republic; University of Salamanca- Institute for Neuroscience of Castille and Leon and Medical School, Spain

## Abstract

We investigated the representation of four typical guinea pig vocalizations in the auditory cortex (AI) in anesthetized guinea pigs with the aim to compare cortical data to the data already published for identical calls in subcortical structures - the inferior colliculus (IC) and medial geniculate body (MGB). Like the subcortical neurons also cortical neurons typically responded to many calls with a time-locked response to one or more temporal elements of the calls. The neuronal response patterns in the AI correlated well with the sound temporal envelope of chirp (an isolated short phrase), but correlated less well in the case of chutter and whistle (longer calls) or purr (a call with a fast repetition rate of phrases). Neuronal rate vs. characteristic frequency profiles provided only a coarse representation of the calls’ frequency spectra. A comparison between the activity in the AI and those of subcortical structures showed a different transformation of the neuronal response patterns from the IC to the AI for individual calls: i) while the temporal representation of chirp remained unchanged, the representations of whistle and chutter were transformed at the thalamic level and the response to purr at the cortical level; ii) for the wideband calls (whistle, chirp) the rate representation of the call spectra was preserved in the AI and MGB at the level present in the IC, while in the case of low-frequency calls (chutter, purr), the representation was less precise in the AI and MGB than in the IC; iii) the difference in the response strength to natural and time-reversed whistle was found to be smaller in the AI than in the IC or MGB.

## Introduction

Behaviorally relevant sounds, such as human speech or species-specific vocalizations, are typically complex sounds characterized by time-varying amplitudes and spectral features. Understanding the principles underlying the processing of communication sounds in the auditory system has attracted great attention due to the behavioral importance of these signals.

Acoustical signals are processed successively in the individual stages of the auditory pathway from the auditory nerve to the auditory cortex. Syka et al. [1] analyzed the responses of neurons in the last two subcortical stages of the auditory pathway, i.e. in the acoustical structures of the midbrain - inferior colliculus (IC) and thalamus - medial geniculate body (MGB) to a set of the most relevant guinea pig vocalizations in ketamine/xylazine anesthetized guinea pigs and found that the vast majority of neurons in both nuclei respond to many calls. The authors also reported different responses to natural and reversed whistle in a portion of IC and MGB neurons. Subsequently, the responses of inferior colliculus neurons to a set of four guinea pig calls were studied in detail [2]. The authors concluded that the spectral and temporal features of the acoustic patterns of vocalizations are, to some extent, encoded in the overall activity of IC units. The results did not confirm the hypothesis that the representation of vocalizations is based on highly specific units selective for a given call. Originally, Creutzfeldt et al. [3] concluded from the results of experiments in non-anaesthetized guinea pigs that the responses of MGB cells represent more components of a call than do those of cortical cells, even if the two cells are synaptically connected. Wallace et al. [4] described the representation of one of the typical guinea pig vocalizations, purr, in the low-frequency thalamic and cortical units in the guinea pig. They reported that the temporal structure of the purr is represented with the same fidelity by the cortical cells as by the cells of the thalamus.

At the cortical level, Wallace and co-workers have described in several studies [4]–[9] the neuronal responses to low-frequency guinea pig calls - purr and chutter. The authors reported a variety of responses to purr: some neurons responded vigorously to many or all of the phrases, but there were also neurons that gave an onset response, responded to a click embedded in the call or did not respond at all. Differences were found between the responses in individual cortical fields [6] and also some differences were reported to exist between the responses from individual cortical layers [8]. Their work, in agreement with the study by Syka et al. [10], demonstrated that the cells in the AI do not exhibit specificity for particular vocalizations, but rather they are sensitive to spectral and/or temporal elements within the call. Similar conclusions were formulated from experiments in cats and monkeys [11]–[13]. Recent work by Pasley et al [14] showed that some attributes of human speech can be reconstructed from population neural activity in the auditory cortex using a linear model based on the auditory spectrogram, but some elements required a nonlinear sound representation based on temporal modulation energy. According to Sadagopan and Wang [15], many neurons in the primary auditory cortex (A1) of awake marmoset monkey are highly selective for complex sound features. Due to their nonlinear spectrotemporal interactions, these neurons may serve as “feature detectors” for a wide range of complex sounds such as marmoset vocalizations. Wang and coworkers (for review see [16]) have shown that neurons in the primary auditory cortex (A1) of the marmoset monkey use a temporal representation to encode slowly varying acoustic signals and a firing rate-based representation to encode rapidly changing acoustic signals. Such encoding represents a progressive transformation in comparison with the coding at the level of the auditory thalamus. The above mentioned findings indicate that the auditory cortex forms internal representations of the temporal characteristics of sounds that are no longer faithful replicas of their acoustic structures. It is thought, therefore, that such transformations are necessary for the auditory cortex to perform a wide range of functions including sound segmentation, object processing and multi-sensory integration.

The aim of the present study was to analyze the responses of A1 neurons to four types of guinea pig calls that represent common sounds of the guinea pig communication call repertoire (there are 11 distinct calls according to Berryman [17]) and that differ fundamentally in their spectral and temporal features [1].

Because responses to identical calls were studied recently in the IC [2] and MGB [18], our goal was also to determine to what extent the responses of A1 neurons are similar or transformed relative to the responses observed in the IC and MGB [19].

## Materials and Methods

### Animal Preparation

The experimental protocol was similar to that reported in [2], [18]. Experiments were performed on 11 adult, healthy, pigmented female guinea pigs weighing 320–500 g. The care and use of animals reported in this study were approved by the Ethics Committee of the Institute of Experimental Medicine ASCR (Prague).

Animals were initially anesthetized with an intramuscular injection of 1 ml/kg of a mixture of ketamine (Narkamon 5%, Spofa) and xylazine (Rompun 2%, Bayer) at a ratio of 2∶1, which corresponds to a dose of 33 mg/kg of ketamine and 6.6 mg/kg of xylazine. Supplementary injections of one-half of the original dose of the ketamine-xylazine mixture were administered every hour to maintain a sufficient level of anesthesia during the experiment. The skin and underlying muscles on the skull were retracted to expose the dorsal cranium between bregma and lambda. A small hole (diameter ∼5 mm) was made by a trephine in the right side of the skull above the auditory cortex, and the dura mater was removed. The animal’s head was rigidly held in a stereotaxic apparatus by a U-shaped holder, which was fixed at its base to the skull by two screws and secured by acrylic resin. This type of fixation enabled the animal’s head to be free for electrode penetration and for free-field acoustical stimulation. A DC-powered electric heating pad maintained a rectal temperature of 37–38°C.

### Acoustic Stimulation

Animals were placed in a soundproof anechoic room, and acoustical stimuli were delivered in free-field conditions from a loudspeaker system (Tesla ARN 5614 and Motorola KSN-1005) placed 70 cm in front of the animal’s head. The acoustic system was calibrated with a B&K 4133 microphone, placed in the position of the animal’s head and facing the speakers. The frequency-response curve was relatively flat and varied by less than ±9 dB between 0.15 and 45 kHz.

**Figure 1:**
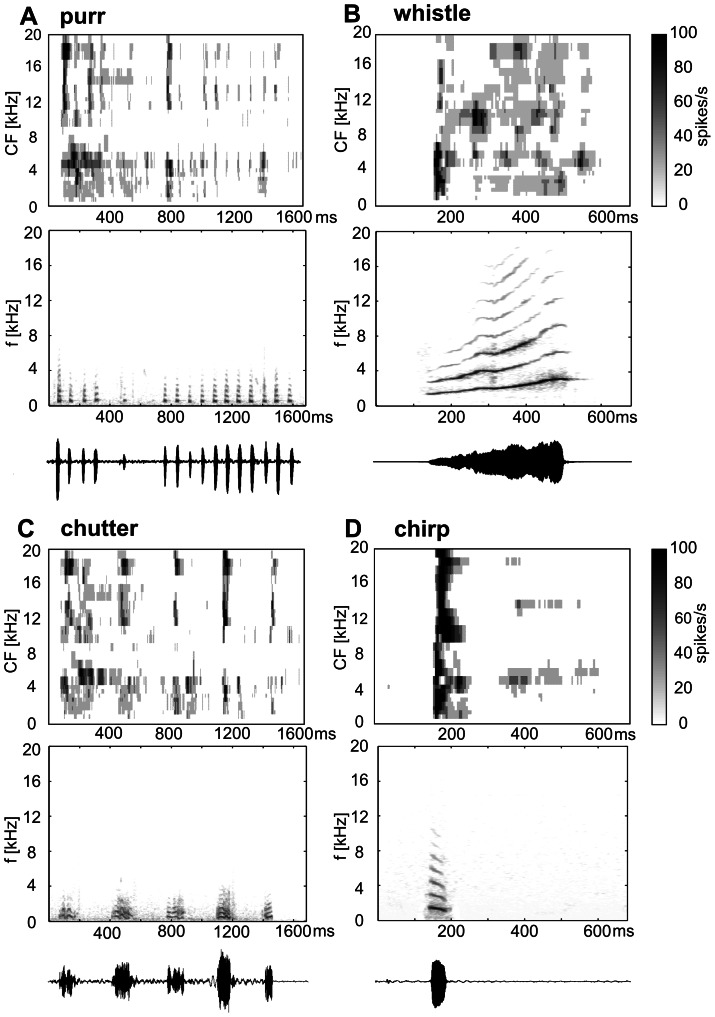
The spectrotemporal response maps of the neuronal population (n = 502, top), the call spectrogram (middle) and the call waveform (bottom) are displayed for purr (A), whistle (B), chutter (C) and chirp (D).

Two types of simple stimuli were used: pure tone pips and broad-band noise (BBN) bursts (both of 100 ms duration with 3 ms rise/fall times presented with a repetition rate of 1 Hz). Four typical vocalization calls (purr, chirp, chutter and whistle – Fig. 1) were chosen from the large repertoire of guinea pig natural calls. Calls were previously tape recorded from spontaneously vocalizing female guinea pigs (age 2–24 months) in a sound-attenuated room [1]. During the experiment digitalized calls (at a sampling frequency of 50 kHz) were played from a PC computer via a TDT System3 setup. The temporal and spectral parameters of the calls and their variability have been described previously in [1]–[2], [20]. One variant of each call was selected for the study, and all animals were stimulated with the same variant of the call. The stimuli were identical to those used in [1]–[2], [18]. Purr is expressed by animals in conjunction with mating behavior and when they seek contact; it consists of a series of regular low-frequency impulses (fundamental frequency ∼300 Hz). Chirp is an isolated brief acoustic impulse with a harmonic structure accompanying the calming of an animal in comfortable conditions. Chutter is a sequence of irregular noise bursts, which accompanies general exploratory activity of an animal and may change to whistle in situations when the animal expresses a feeling of separation or wants to attract the attention of its caretaker. Whistle is a long-lasting frequency- and amplitude-modulated sound consisting of many harmonics over a wide frequency range. The stimuli were presented once every 2.9 s for chutter and purr, 2.5 s for chirp and 2.2 s for whistle.

Because vocalizations vary in intensity over time, the call intensity was characterized by the peak value of the sound level measured by the B&K 2231 sound level meter. The responses reported in this paper were obtained at the peak value of 75 dB SPL. The time-reversed whistle was generated by reversing the time course of the natural whistle call. All other parameters of the whistle, such as the sound level and the repetition rate, were preserved.

The features of the neuronal responses were compared not only to the original calls, but also to calls corrected for the auditory threshold of the guinea pig. Such ‘corrected’ calls were generated using a filter mimicking the guinea-pig audiogram (Table S1) for the processing of the original calls. Such ‘corrected’ calls were not used for stimulation, but only for analysis.

### Recording of Neuronal Activity

The activity of neurons in the auditory cortex was recorded with 16-channel multi-electrode probes from NeuroNexus Technologies (four shank, each of 4 electrode tips, site spacing 50 µm, shank spacing 125 µm). The electrode was inserted into the AI using an electronically controlled microdrive with 1 µm steps. As a search stimulus, BBN bursts or tone sweeps were used.

The signal from the multi-electrode was processed by a TDT System3 setup connected to a PC computer running the BrainWare program, where the activity was saved and later analyzed. Spike sorting based on the waveform shape was performed offline in the BrainWare program. As a rule, only the activity from reliably discriminated single units was included in the data set.

The position within AI was identified on the basis of stereotactic coordinates and the functional properties of the neurons; in particular, the tonotopic organization and short latency of the response to tonal stimuli were taken into account [6]. Also helpful were the pattern of the cerebral arteries and an evaluation of the responses to white noise.

### Data Analysis

The following parameters of the response were evaluated in the process of data analysis:

The characteristic frequency (CF) was determined as the frequency of a pure tone stimulus that evokes a neuronal response at a minimal intensity. An automatic routine for presenting tone bursts of varying intensity (5 dB step) and frequency (0.2 oct. step) in a pseudo-random order was used to obtain the frequency response area from which the CF value was identified.

The level of the spontaneous firing rate of each neuron was evaluated from 600 ms periods preceding the onset of a tone-burst stimulus at near-threshold intensity and was calculated as the total number of spikes normalized per 1 second. In several guinea pigs, long recordings (3–5 min) of spontaneous activity were made and the rate was calculated. Spontaneous activities calculated from inter-stimulus periods and from long-term recordings were not significantly different. The driven firing rate was expressed as the total number of spikes over the stimulus duration, shifted by the response latency and normalized per 1 stimulus and per 1 second with the spontaneous firing rate subtracted.

The vector strength (VS), describing the synchronization of the neuronal response to the periodicity of the stimulus, was calculated according to [21]–[22] for two sounds consisting of several phrases: chutter and purr. The values of the VS were evaluated from the entire response to chutter (i.e., from the response to all 5 phrases of this sound) and from the response to the last 11 phrases of purr. The significance was evaluated using Rayleigh statistics [23]–[24].

Individual PSTHs (peristimulus time histograms; bin width 5 ms) were sorted according to the CF and average PSTHs were calculated from all PSTHs with a CF belonging to the particular CF bin (with a 0.5 kHz bin size). Then the averaged PSTHs were aligned according to the CF (Y axis) to create a 3D plot of the spectrotemporal response map illustrating the response pattern of the neuronal population to particular calls. The correlation between PSTHs (either individual or averaged, 5 ms bin) and the sound envelope of the appropriate call was calculated using a 700 ms window for whistle and chirp and a 1,700 ms window for chutter and chirp.

A rate vs. CF profile was calculated as the average driven rate [25] of all units having a CF within a 0.35 octave-wide frequency window moved in 1/8 octave steps. Only points calculated from three or more units were included into the profile. The response was calculated from a time window shifted by 15 ms relative to the call to compensate for the response latency.

All statistical tests were performed using Prism software.

## Results

Recordings from 502 neurons were collected in 11 guinea pigs. The CF was evaluated for all units responding to pure tones and ranged between 0.3 kHz and 26 kHz (Fig. S1). Neurons typically responded to many calls (62% responded to all 4 calls and only 2% gave a significant response to just one call). A comparison of the spectrograms of the calls with the response patterns of the neuronal population, illustrated by the spectrotemporal response maps (Fig. 1 A–D) that were constructed from all PSTHs of individual units, indicated to what extent the neuronal response is driven by the spectrotemporal pattern of the vocalization calls. The gray scale reflects the average response strength of all recorded units of CFs within a 0.5 kHz wide frequency band at a particular time (5 ms bin). Visual inspection of the spectrotemporal response maps revealed that the patterns present in the population response maps correspond with many features of a calĺs acoustical pattern, but also demonstrated some striking differences. An example of dissimilarity was the occurrence of the response of high-CF neurons to purr, chutter, chirp and to the initial part of the whistle (140–250 ms), even though there is no energy present in this frequency range. Another difference was evident when the spectrotemporal response map to chutter (Fig. 1C) was compared with the spectrogram of this call: the duration of the response to individual bouts of the call was shorter than the duration of the bouts. The spectral and temporal aspects of the encoding are analyzed separately in the following paragraphs.

### Representation of Temporal Features

**Figure 2:**
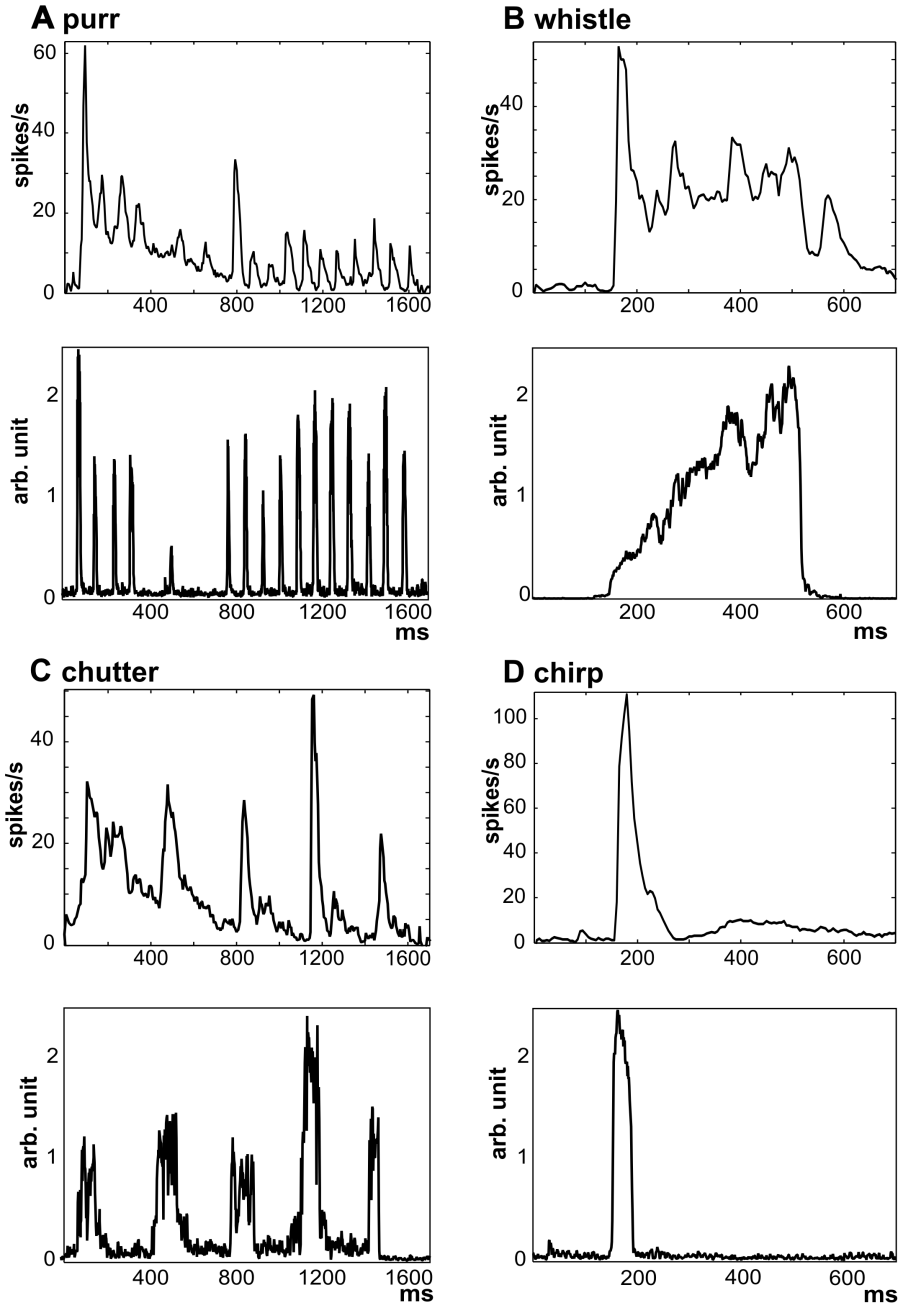
Comparison of the temporal envelope of the calls and the neuronal response. A comparison of the population PSTHs (n = 502, top) and the sound envelopes (bottom) is shown for all four calls: purr (A), whistle (B), chutter (C) and chirp (D). Each population PSTH is calculated as the average PSTH of all recorded units.

**Figure 3:**
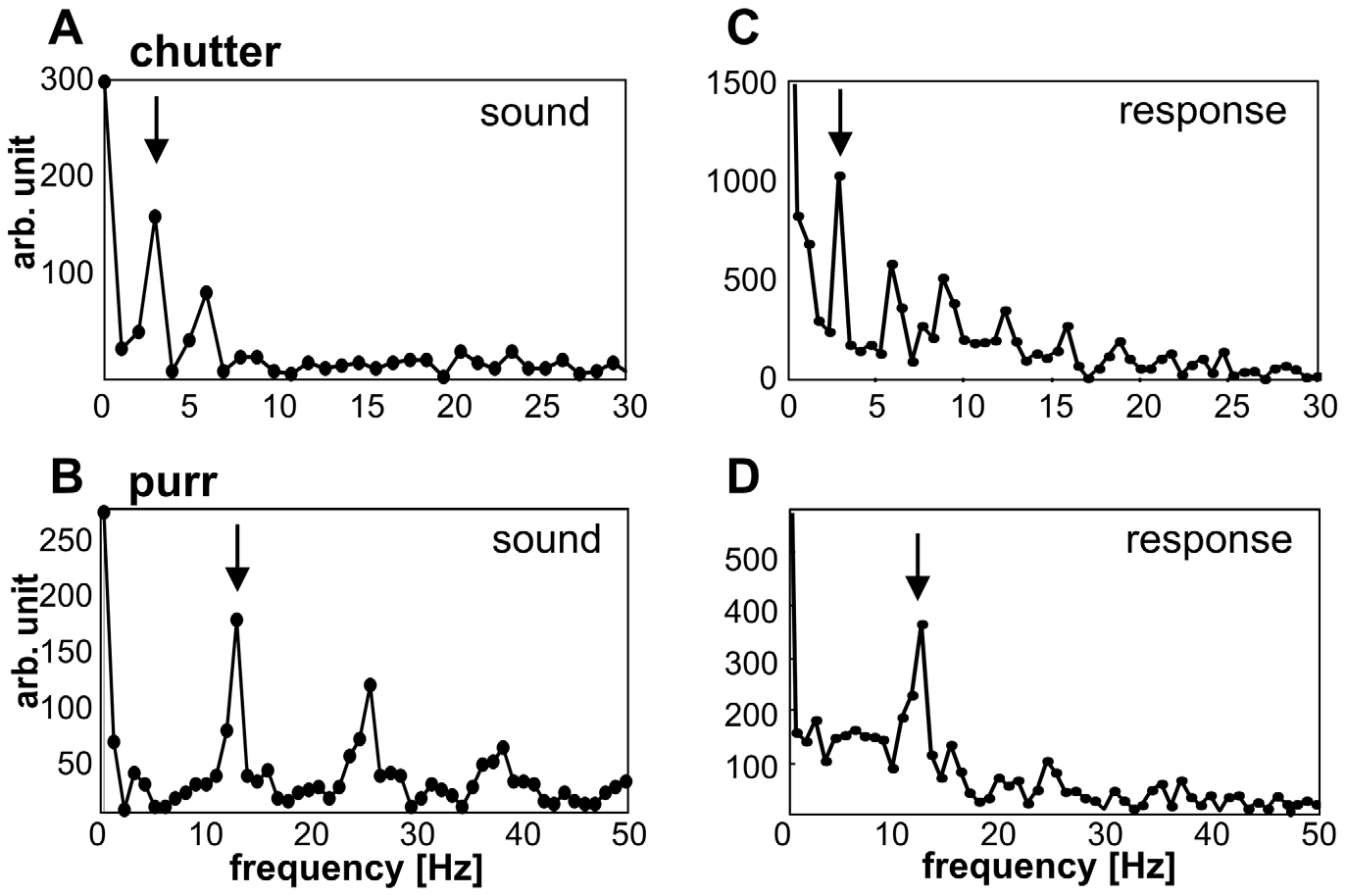
Coding of the vocalization frequency for chutter (A, C) and purr (B, D). The local maximum in the spectrum of the sound envelope (marked by an arrow in A and B) that corresponds to the average repetition rate of phrases can be detected in the frequency spectrum of the population PSTH (marked by an arrow in C and D) for chutter (C) and purr (D).

Purr and chutter are characterized by a rhythmic repetition of several phrases (Fig. 1 A,C, Fig. 2A,C). The repetition rate of the individual phrases (i.e., the vocalization frequency, Fig. 3A,B) was indicated by the first peak in the frequency spectrum (Fig. 3C,D) calculated from the population PSTH (Fig. 2C, D) in the case of chutter (Fig. 3A,C) as well as of purr (Fig. 3B,D). The synchronization of the neuronal firing to the vocalization frequency was also detectable in the PSTHs of individual units (Fig. 2): 32% of units showed significant synchronization to purr (Rayleigh statistics of the VS, P<0.001) and 56% to chutter. Synchronization to these vocalization stimuli was more frequent in low-CF units: 62% of units with a CF below 5 kHz were significantly synchronized to purr and 73% to chutter. The neuronal activity showed various levels of adaptation in the repetitive firing of the responses, with a decline in the response during the presentation of purr. Similar variability in the decline of the response during the later phrases of a call was observed for chutter.

Population PSTHs of AI neurons reflected the sound temporal envelopes, as demonstrated in Fig. 2. When the similarity between the sound envelope and the population PSTH (n = 502, Fig. 2) was evaluated using a correlation coefficient (r), a lower correlation was found for purr (r = 0.44) than for chutter (r = 0.56). The correlation coefficient in the case of purr was lowered by a decrease in the neuronal response during the purr presentation even if the amplitudes of the individual peaks of purr did not decline. In the case of chutter, correlation was limited by the onset character of the activity, which is apparent from the much shorter response than the duration of the individual phrases of chutter (Fig. 2C). The short call chirp evoked a short burst of activity, which corresponds well with the shape of the sound envelope (Fig. 2D, r = 0.95). A lower correlation was present in the case of whistle – a sound of longer duration (r = 0.62). The dominant element in the response of whistle was the onset response (Fig. 2B), which contrasts with the weak energy at the beginning of the whistle. The strong onset reaction can be particularly surprising for neurons with higher CFs (>8 kHz), because there is almost no energy of the sound in the appropriate frequency range, but this aspect is already present at the MGB level [18]–[19].

### Responses to Whistle and Time-reversed Whistle

**Figure 4:**
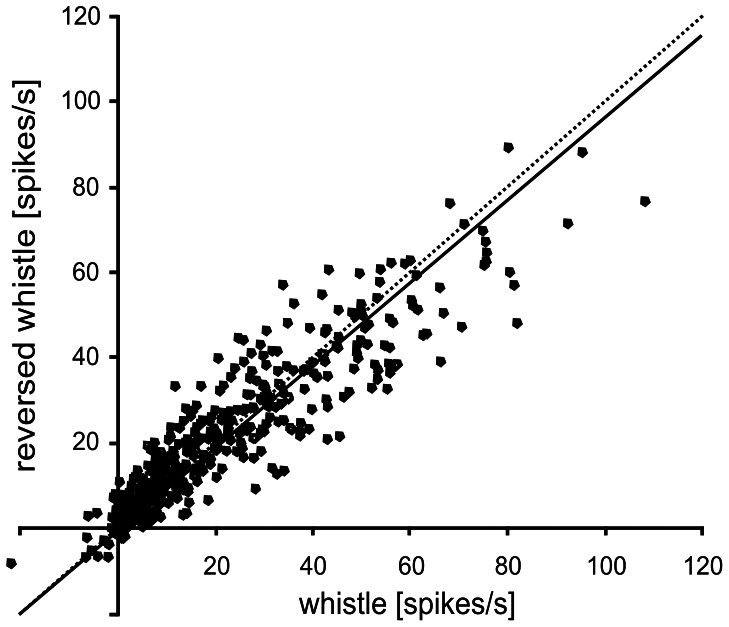
Relationship between the response to whistle and the response to time-reversed whistle in individual neurons (n = 502). Each dot represents one unit. The slope of the regression line (solid line) is not significantly different from one (dashed line), P>0.05.

The response to stimulation by a time-reversed form of whistle was evaluated and compared to ‘natural’ whistle. Because the reversal of the calĺs time course changes only the call’s temporal pattern but the call spectrum remains unchanged, a comparison of the responses to the 'natural' and time-reversed call may indicate the selectivity of the neuronal responses for the temporal pattern of the call. Fig. 4 shows a comparison of the responsiveness to the natural and reversed whistle measured by the driven rate. The driven firing rates obtained for whistle and reversed whistle in the same unit were positively correlated (R^2^ = 0.83). The slope of the regression line was 0.96, which means that in the neuronal population the average response to the time-reversed whistle was slightly (by 4%, on average) weaker than to the whistle. However, the difference was not significant (the slope of the regression line does not differ significantly from one; p>0.05).

### Representation of Spectral Features

The spectrotemporal response maps for individual calls (Fig. 1) were not just copies of the call spectrogram, even though there are apparent similarities between the pattern of the firing rate of the units and the spectral composition of the call. To compare the spectral features of the calls and neuronal firing, the neuronal representation of the spectral features was analyzed using rate-CF profiles, which were compared to the short-term sound spectrum. The rate-CF profile shows the average driven rate of all neurons having a particular CF - neurons were grouped according to the value of the CF and then the average firing rate was calculated within each group.

**Figure 5:**
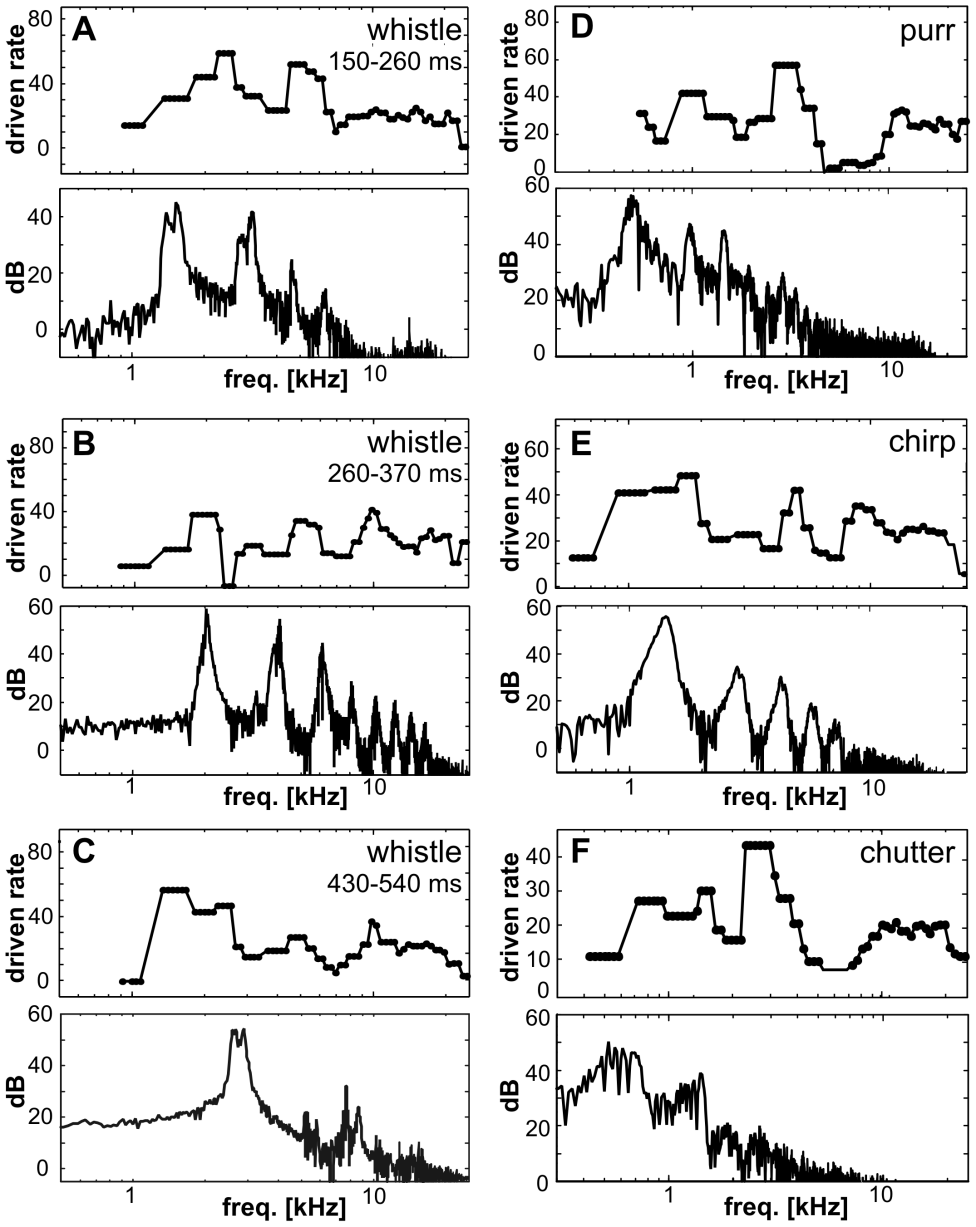
Comparisons of the rate-CF profiles (n = 502, top) and call short-term spectra (bottom) for three consecutive parts of whistle (A–C – the appropriate time interval is indicated above every rate-CF profile), for purr (D – data calculated over the first phase containing four elementary phrases), for chirp (E) and for chutter (F – calculated over the first phrase of the call).

In Fig. 5, rate-CF profiles are shown for three consecutive parts of whistle (A–C), for purr (D), chirp (E) and chutter (F). Fig. 5A shows the situation for the initial part of whistle (the first 110 ms of the call). The correlation coefficient between the short-term sound spectrum and the appropriate rate-CF profile was 0.48, and the correlation between the ‘corrected’ short-term sound spectrum (the correction reflects the shape of the guinea pig audiogram) and the rate-CF profile was 0.46. The rate-CF profile indicated the dominant spectral component of the fundamental frequency and the 1st harmonic (1–4 kHz), but these two frequencies were not separated. A second peak appeared in the range of 5–7 kHz, and there is also some activity at higher frequencies, even though there is almost no energy in the sound above 8 kHz at this time. Fig. 5B shows a different situation, which represents the middle part of whistle; the correlation coefficient was 0.12 for the call spectrum and 0.08 for the ‘corrected’ spectrum. The dominant fundamental, 1st and 2nd harmonic frequencies were only weakly expressed in the elevated firings of neurons with corresponding CFs. Higher harmonic frequencies were poorly reflected in a flat and weak rate profile. Fig. 5C represents the late part of whistle when the dominant spectral component is the fundamental frequency of ∼3 kHz. The rate-CF profile showed only a slightly elevated activity of neurons with CFs of ∼1–3 kHz followed by an almost flat profile (Figs. 2B); the correlation coefficient was 0.16 for the call spectrum and 0.05 for the ‘corrected’ spectrum, respectively. In the case of chirp (Fig. 5E), the value of the fundamental frequency corresponds to the position of the first maxima in the rate-CF profile, but the harmonic frequencies are not clearly expressed; the correlation coefficient was 0.32 for the sound spectra and 0.41 for the ‘corrected’ sound spectra. In the rate-CF profiles of the two low frequency calls – chutter and purr (Fig. 5D,F) – some local spectral peaks were intensified and created dominant elements, which resulted in low correlation coefficients between the sound spectra and the rate-CF profiles (purr: 0.13, chutter: 0.16) as well as between the ‘corrected’ sound spectra and the rate-CF profiles (purr: 0.07, chutter: 0.12).

### Differences in the Response Patterns among Neurons

As suggested by Fig. 1, there were differences in responses among neurons, in particular due to different CFs. The neuronal response patterns to chirp were highly correlated with the sound envelope and quite homogeneous among neurons: the mean correlation coefficient between sound envelope and response pattern of individual neurons was 0.72±0.18 (the mode 0.85). The response patterns of low-CF and high-CF neurons were similar, only the response of high-CF neurons was typically weaker (Fig. 5A). In contrast to chirp, in case of whistle were neuronal patterns less correlated to sound envelope and more variable (0.32±0.24, the mode 0.15). Also high-CF neurons differed significantly from low-CF neurons in their response to whistle (Fig. 5B) due to the complex spectrogram of whistle. For the other two sounds – purr and chutter correlation coefficients between sound envelope and response pattern of individual neurons were 0.22±0.24 (the mode 0.37) and 0.24±0.21 (the mode 0.34).

However, the CF is not the only feature determining the neuronal response. Fig. 5C shows the average response pattern to whistle obtained in neurons with a high correlation between the neuronal pattern and the sound envelope of whistle and also those having a negative value of the correlation coefficient. Neurons producing a negative correlation fired spikes at the beginning of the whistle (first peak), at the beginning of the high frequency component of the whistle (the second peak) and at the end of the call. This means that the neuronal response of these neurons identified some events in the call (beginning, end) but did not carry information about the shape of the sound envelope.

## Discussion

### Representation of Temporal and Spectral Features

The results of this study demonstrate that AI neurons typically respond to many calls with a response pattern that generally reflects the basic spectrotemporal features of the call. Neuronal responses were time-locked to one or more temporal elements of the calls. The correlation coefficient between the sound envelope and the population response pattern differed among individual calls: maximal correlation was observed in the case of chirp (a call with an isolated short phrase), less correlated activity was found in the case of longer calls (chutter and whistle) or purr - a call consisting of short phrases with a fast repetition rate of phrases. Differences in correlation are apparently related to the temporal features of the calls – namely the duration of the call’s phrases and their repetition rate. The phasic character of neuronal responses in the AI of anesthetized animals, when the response is concentrated on a particular phase of the sound (mostly on the beginning of the call’s phrase - an ‘on’ response) with weak or no sustained firing, maintains a strong similarity between stimulus and response for short calls but reduces the correlation for longer stimuli. The modulatory effect of anesthesia on neuronal activity must be considered as an important factor. Astl et al [26] reported that the type of anesthesia is also important for the neuronal response in the guinea pig: the occurrence of neurons with an onset type of response in the IC was significantly larger with pentobarbital than with ketamine or urethane anesthesia. Specifically, the effect of ketamine was analyzed in our study of cortical activity [10]; we found a non-uniform effect of the anesthesia on neuronal activity – the anesthesia mostly, but not exclusively, suppressed the neuronal responses and also modulated the neuronal pattern. The second factor is represented by the repetition rate of phrases within a call. It is known that there exists a decrease in the highest modulation frequency that influences the neural response in the auditory cortex relative to lower centers of the auditory pathway [27]. A fast repetition rate of a phrase may result in a lack of neural activity synchronized to the amplitude modulation of the stimulus. This general principle was observed by using a variety of natural calls by Creutzfeldt et al. [3], who reported that in cortical cells of the guinea pig, the repetitive elements of a call were not represented if the repetition rate was too high. Several types of responses of low-frequency neurons to purr were described by Wallace et al [6]. They found neurons in the AI of the guinea pig that responded vigorously to many or all of the phrases, neurons that gave only an onset response as well as neurons that did not respond at all, which may reflect the fact that AM phase-locking in cortical neurons varies over a wide range [26]. Non-uniform neuronal response patterns and their modulation by anesthesia were reported by Syka et al. [10].

A relatively poor rate representation of the sound spectrum was found at the cortical level, even though the response patterns demonstrated a strong dependence of the firing pattern of the units on the spectral composition of the call. Particularly interesting is the observation of high frequency neurons responding during the presentation of calls in which there is no or little energy present at those frequencies. A similar observation of high-CF neurons responding to low frequency calls was already made at the subcortical level – in the IC (e.g., Figs. 4 and 5 in [2]) and MGB (Figs. 1 and 5 in [18]). The origin of the surprising response in high-CF neurons was also tested in the MGB by stimulating high-CF neurons with a low-pass or high-pass filtered whistle (Fig. 5C–E in [18]). An early response (∼150–200 ms) was present in the reaction of high-CF neurons to whistle as well as in the reaction to the low-frequency component of whistle, but was completely absent in reaction to the high-frequency component of whistle, having almost no energy at that time. This comparison demonstrates that the early response is influenced by the low-frequency component of whistle, even though this frequency band is well below the neuronal CF. A similar effect was also reported in other studies, e.g., Holmstrom et al. [28] reported that the CFs of the IC neurons in mice that responded to the natural female upsweep were clustered around 15 kHz despite the high spectral content (82–98 kHz) of this vocalization. Our findings thus correlate well with the data obtained by Nagarajan et al. [13], who reported that in contrast to the temporal envelope of complex vocalizations, the spectral envelope of a call is poorly represented in the mean-spectral representation of AI responses in anesthetized common marmosets.

It is assumed that heterogeneity among neurons contributes to coding efficacy in the central auditory system. In most of the analyses, we treated the neurons as a single population, but one can expect differences among neurons according to the CF, recording depth, cell type, temporal response pattern, monotony of the rate-level function and/or other factors that may significantly influent the way neurons respond to different vocalizations. In contrast to this, some parameters that predict the response to simple stimuli (e.g., tones) well may fail in the case of complex stimuli such as vocalization calls. We already mentioned the CF and its only limited power to predict the response to calls. Another example could be rate/level monotony, because already in the dorsal cochlear nucleus, the principal cell shows fundamentally different rate-level functions in response to narrow-band stimuli (tones) in comparison to wide-band stimuli.

In our study we used the CF as the primary parameter determining the neuronal responses. The heterogeneity of the neuronal response patterns is demonstrated to a different extent for the individual calls. While the neuronal responses (individual PSTHs) to chirp are quite homogeneous (and similar to each other), the neuronal responses are much more heterogeneous in the case of whistle, where the response pattern depends on the CF due to the complex spectrogram of the call.

The study by Wallace et al. [4] showed that within a cortical column most units gave a similar response to purr and reported no correlation between the type of response and the cortical depth. In a more recent work [8], the authors described that the responses to two vocalizations varied between layers, specifically layers II/III vs. layers V/VI. Our recordings were typically performed at a depth corresponding to layers II-IV, and in accordance with these studies we did not observe any apparent variability in the response patterns with cortical depth.

### Transformation of the Neuronal Responses from the IC to the MGB and Further to the AI

**Figure 6:**
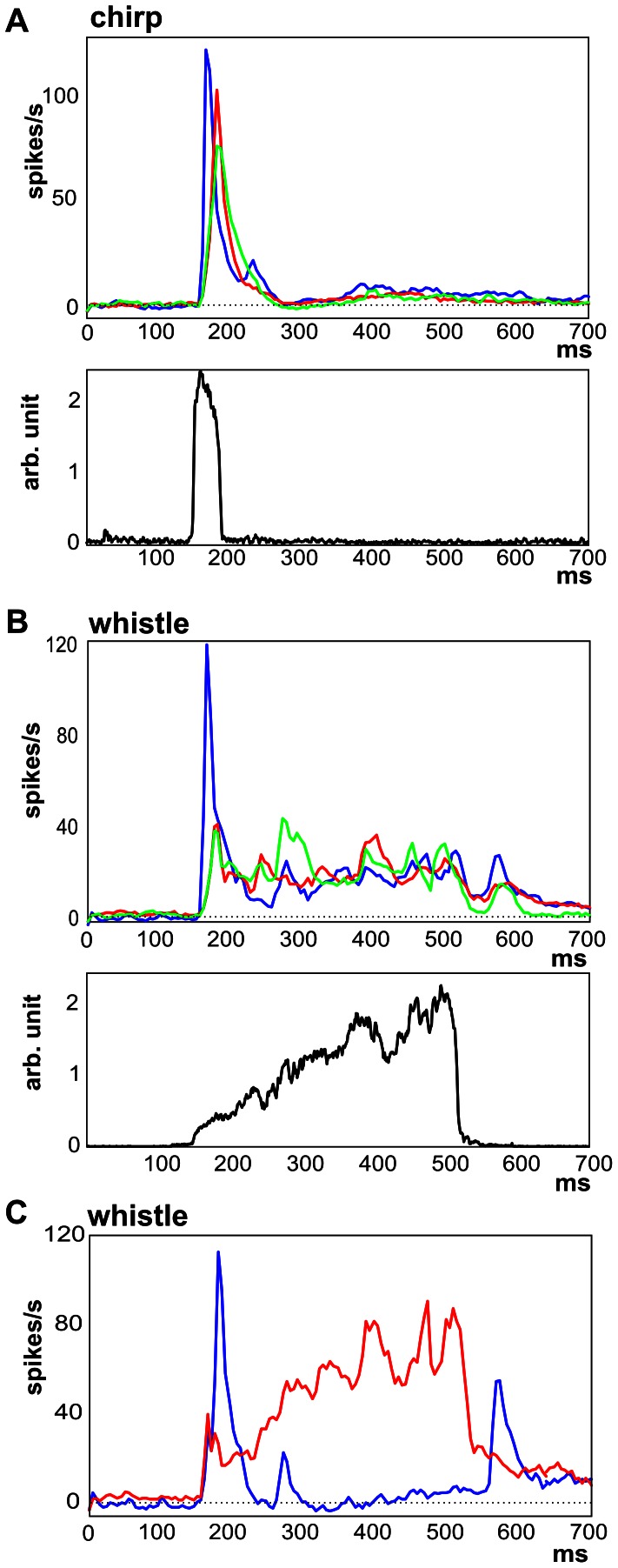
Differences in neuronal response patterns. The subpopulation PSTHs calculated for all low-CF neurons (CF <7 kHz, blue), middle-CF (7–14 kHz, green) and high-CF (>14 kHz, red) subpopulation PSTHs are compared for chirp (A) and whistle (B). The lower panels of (A) and (B) shows the call envelopes. Plot (C) shows a comparison of the subpopulation PSTHs calculated from individual PSTH with negative correlation to sound envelope (blue) and subpopulation PSTHs calculated from individual PSTH with high correlation to sound envelope (r>0.5, red) for whistle.

The stimuli used in the present study were identical to those used in previous studies in the IC [2] and MGB [18], allowing us to make a direct comparison of the results from three consecutive levels of the guinea pig’s auditory pathway: IC, MGB and AI. Transformation of the neuronal responses from the IC to the MGB and further to the AI is apparent also in the responses to simple sounds. While in ketamine anesthetized guinea pigs about ¾ of the neurons in the IC have sustained response patterns to a tone [29], in the MGB the portion of sustained response patterns is below ¼ [30]. Also, a comparison of the representation of the temporal features of individual vocalization signals clearly shows that neuronal responses are transformed when propagated from the IC to the MGB and further to the AI, but to a different extent for individual calls. The neuronal responses to chirp (which has the character of an isolated short pulse) are fully comparable in all three stages (a comparison of the correlation coefficients in the IC, MGB and AI is shown in Fig. 6A), and therefore no transformation of the response is apparent when information propagates from the IC to the MGB and then to the AI. In the case of chutter and whistle, the correlation between the sound envelope and the population PSTH in the AI remains similar as in MGB neurons, which is lower than in the case of IC neurons (Fig. 6A). This finding suggests that the transformation of these call representations occurs during the transition from the IC to the MGB and then is preserved in the AI. Purr is the only call for which responses in the IC and MGB are very similar and then the response pattern is modified in the AI: the neuronal response to purr in the AI shows a decrease in the correlation with the call in comparison with the MGB. This is caused by a more pronounced decrease of the response in the AI during the duration of the stimulus.

All of these findings demonstrate that all communication calls are not processed in the same way by neuronal circuits of the auditory pathway and that there is not a unique pattern of communication call processing. An important role in the processing of vocalizations in the auditory system is played by the acoustical patterns of the calls, which may vary markedly among the calls of a species-specific vocalization repertoire.

Creutzfeldt et al. [3] concluded from the results of experiments in non-anaesthetized guinea pigs that the responses of MGB cells represent more components of a call than do those of cortical cells, even if the two cells are synaptically connected. In cortical cells, the repetitive elements of a call were not represented if the repetition rate was too high. Wallace et al. [4] reported that low-frequency neurons with the CF below 1.1 kHz in the ventral division of the MGB in the guinea pig gave a more faithful representation of the multiple phrases of purr than cells in the AI, but overall the thalamic (including the dorsal, ventral and medial divisions) and cortical populations seemed to have a similar range of correlation values.

**Figure 7:**
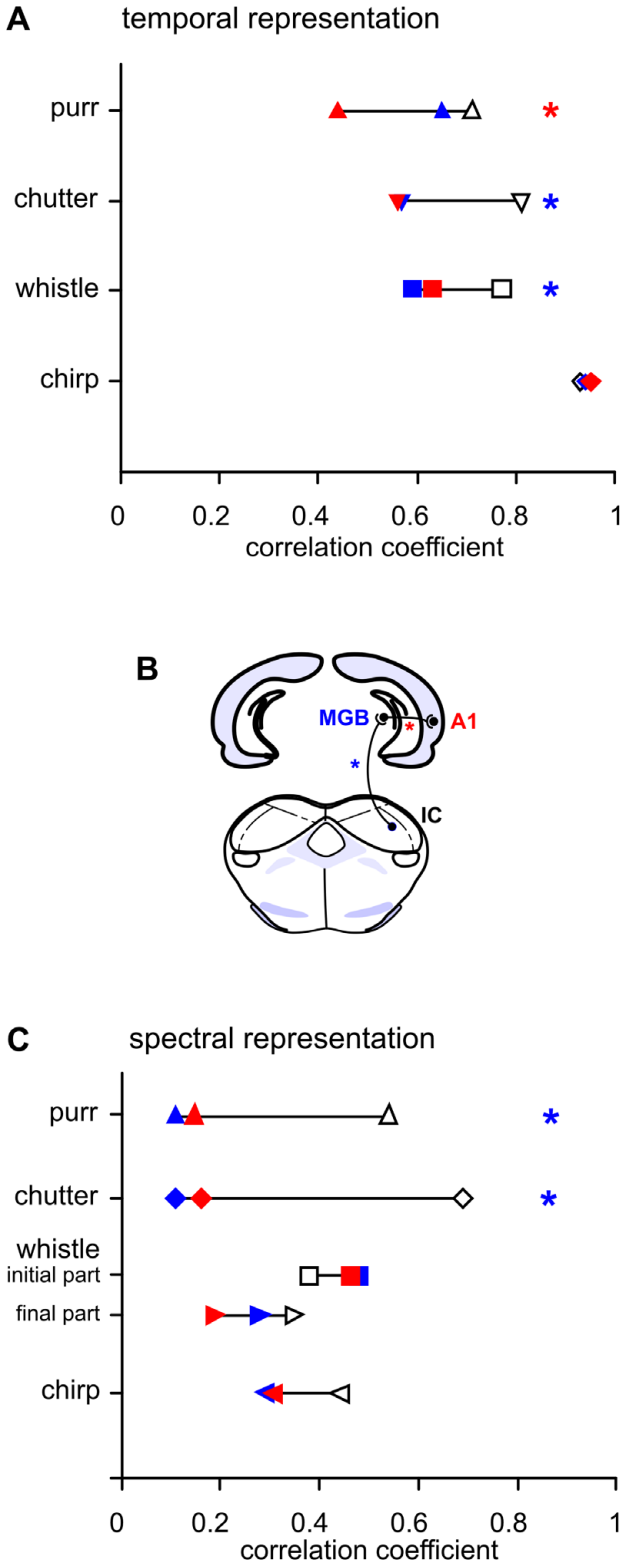
Comparison of subcortical nuclei (IC, MGB) and the auditory cortex. The correlation coefficients between the sound envelope and the averaged PSTH (A) and the correlation coefficients between the sound frequency spectrum and rate vs. CF profile (C) are compared in the inferior colliculus (IC, open), medial geniculate body (MGB, blue) and auditory cortex (AI, red) for individual call. Panel (B) shows a schematic drawing of a part of the ascending auditory pathway. IC data based on 153 neurons are taken from [2]; MGB data calculated from 209 neurons are taken from [18]. The bootstrap method was used to determine whether the values of the correlation coefficients in individual nuclei were statistically different (P<0.01). The blue stars indicate cases of tecto-thalamic transformation of the neuronal response in which the IC data were significantly different from the MGB and AI data, while the difference between the MGB and AI was not significant. The red star indicates a case of thalamo-cortical transformation of the neuronal response in which the AI data were significantly different from MGB and IC data, while the difference between the MGB and IC was not significant.

The relatively poor rate representation of the sound spectrum that was found at the cortical level corresponds to previous studies in the IC [2] and MGB [18], where a limited rate representation of the call was observed as well. A direct comparison of the AI with the IC and MGB data (Fig. 7B) suggests that the representation of the spectral features found at the level of the MGB is also preserved in the AI. Therefore, for wideband calls (whistle, chirp) the rate representation of the call spectra is preserved in the MGB and AI at the level present in the IC, while in the case of low-frequency calls (chutter, purr), the representation is less precise in the MGB and AI than in the IC.

### Natural vs. Time-reversed Calls

Many studies have reported a preference of neuronal responses for natural calls over artificial, time-reversed ones, in the cortex of species such as songbirds [31]–[32], bat [33], cat [12] or primates [11], [34]. The weaker response to temporally manipulated calls is often thought to be attributable to their lack of behavioral relevancy. Our data displayed only a very weak preference of cortical responses to whistle over time-reversed whistle, which was not statistically significant. Also Huetz et al. [35] found in guinea pig only few thalamic and cortical cells displaying a firing rate preference for the natural version of vocalizations, but suggested importance of the temporal discharge pattern. Philibert et al. [36] concluded that the thalamic neurons of guinea pigs did not exhibit a significant preference for the natural over the time-reversed versions of the calls. In our study in the MGB [18], a comparison of the responses to whistle and time-reversed whistle showed that whistle evoked a slightly stronger response in MGB neurons, on average, than did the artificial (time-reversed) whistle - a difference of 13%. A preference for the natural sound was also previously found in the IC [2], where the response difference between these two sounds was even more pronounced (24%).

The similarity of the response strengths to whistle and time-reversed whistle would apparently contrast with the well known experience of a lack of understanding in the case of time-reversed speech in humans. However, because the response pattern of the neuronal population reflects the basic spectrotemporal patterns of the calls, there is the possibility that processing is based on the spectrotemporal response pattern of the neuronal population rather than on simply the value of the response strength. Some studies (e.g. [37]) have suggested that there are some identified associated auditory cortical areas with a greater involvement in the processing of communication calls that may contain a greater proportion of cells that respond preferentially to communication calls over artificial and simple stimuli. Because we focused on the AI in our study and no recording was made from the auditory association areas, the data do not allow us to discuss this aspect.

In summary, this study describes the neuronal representation of a set of guinea pig vocalization sounds in the AI and thus enables a direct comparison with data obtained in similar experiments in the IC [2] and MGB [18]. Our data demonstrate various levels of fidelity in temporal envelope representation – highly accurate in the case of a rapidly modulated temporal structure and/or a lower repetition rate, but less precise for slow amplitude modulation and/or a quick repetition rate. As regards the representation of the call spectrum, the spectral features in the AI are preserved at the level of the MGB. The difference in the response strength to natural and time-reversed whistle was found to be smaller than that in the IC and MGB. In conclusion, we have found specific transformations of the response patterns from the level of the IC to the level of the AI for individual calls.

## Supporting Information

Figure S1 **Distribution of the neuronal characteristic frequencies (CFs).**
(DOCX)

Table S1 **Parameters of the filter mimicking the guinea-pig audiogram.**
(DOCX)
