# Supplementary material for: Cortical Representation of Species-Specific Vocalizations in Guinea Pig
Source: PLoS One. 2013 Jun 13;8(6):e65432. doi: 10.1371/journal.pone.0065432 (PMC3681779; doi:10.1371/journal.pone.0065432)
Supplement: Figure S1 — Distribution of the neuronal characteristic frequencies (CFs). (DOCX) [file pone.0065432.s001.docx]

**Supplementary Figure S1. Distribution of the neuronal characteristic frequencies (CFs).** The sampling of neuronal CFs covers the frequency range of all calls.
