# Supplementary material for: Cortical Representation of Species-Specific Vocalizations in Guinea Pig
Source: PLoS One. 2013 Jun 13;8(6):e65432. doi: 10.1371/journal.pone.0065432 (PMC3681779; doi:10.1371/journal.pone.0065432)
Supplement: Table S1 — Parameters of the filter mimicking the guinea-pig audiogram. (DOCX) [file pone.0065432.s002.docx]

**Supplementary Table S1. Parameters of the filter mimicking the guinea-pig audiogram.** The parameters are almost identical to audiograms reported by Heffner R et al. (1971) JASA 49:1888-1895; Syka J & Popelář J. (1980) Hear Res 3: 205-213 or Gourévitch B et al. (2009), Brain Res 1304: 66-79,
